# Longitudinal liquid biopsy anticipates hyperprogression and early death in advanced non-small cell lung cancer patients treated with immune checkpoint inhibitors

**DOI:** 10.1038/s41416-022-01978-1

**Published:** 2022-09-29

**Authors:** Elisabetta Zulato, Paola Del Bianco, Giorgia Nardo, Ilaria Attili, Alberto Pavan, Andrea Boscolo Bragadin, Ludovica Marra, Giulia Pasello, Matteo Fassan, Fiorella Calabrese, Valentina Guarneri, Pier Franco Conte, Stefano Indraccolo, Laura Bonanno

**Affiliations:** 1 Basic and Translational Oncology Unit, Istituto Oncologico Veneto IOV IRCCS, Padova, Italy; 2 Clinical Research Unit, Istituto Oncologico Veneto IOV IRCCS, Padova, Italy; 3 Department of Surgery, Oncology and Gastroeneterology, Università degli Studi di Padova, Padova, Italy; 4 Immunology and Molecular Oncology Diagnostics Unit, Istituto Oncologico Veneto IOV IRCCS, Padova, Italy; 5 Medical Oncology 2, Istituto Oncologico Veneto IOV IRCSS, Padova, Italy; 6 Surgical Pathology Unit, Department of Medicine, Università degli Studi di Padova, Padova, Italy

**Keywords:** Non-small-cell lung cancer, Tumour biomarkers

## Abstract

**Background:**

Immune checkpoint inhibitors (ICIs) have revolutionised treatment of advanced non-small cell lung cancer (aNSCLC), but a proportion of patients had no clinical benefit and even experienced detrimental effects. This study aims to characterise patients experiencing hyperprogression (HPD) and early death (ED) by longitudinal liquid biopsy.

**Methods:**

aNSCLC receiving ICIs were prospectively enrolled. Plasma was collected at baseline (T1) and after 3/4 weeks of treatment, according to the treatment schedule (T2). Cell-free DNA (cfDNA) was quantified and analysed by NGS. cfDNA quantification and variant allele fraction (VAF) of tumour-associated genetic alterations were evaluated for their potential impact on outcome. The genetic alteration with the highest VAF (maxVAF) at baseline was considered as a reference.

**Results:**

From March 2017 to August 2019, 171 patients were enrolled. Five cases matched criteria for HPD and 31 ED were recorded; one overlapped. Quantification of cfDNA at T2 and its absolute and relative variation (T2–T1) were significantly associated with the risk of ED (*P* = 0.012, *P* = 0.005, *P* = 0.009). MaxVAF relative change (T2–T1/T1) was significantly associated with the risk of HPD (*P* = 0.02). After identifying optimal cut-off values, a two-step risk assessment model was proposed.

**Discussion:**

Liquid biopsy performed early during treatment has the potential to identify patients at high risk of ED and HPD.

## Introduction

Immunotherapy is widely considered one of the most important advancements in the treatment of advanced non-small cell lung cancer (aNSCLC). While the majority of non-oncogene addicted aNSCLC patients are currently treated with immune checkpoint inhibitors (ICIs) either in monotherapy or in combination with chemotherapy [1–5], great heterogeneity in response and duration of clinical benefit has been observed. The search of predictive biomarkers is one of the main burning issues in thoracic oncology, and, at present, the only available predictive marker for ICIs is PD-L1 expression in tumour cells, although clearly showing its limitations in the clinical setting.

Notably, there is increasing evidence that ICIs may be associated with very poor outcome or even detrimental effects in a quote of NSCLC patients. This concept was initially related to the observation of an increased number of deaths recorded during the first 12 weeks in patients receiving ICIs versus chemotherapy [6]. Furthermore, a specific radiological pattern of progression, called hyperprogression (HPD), has been associated to the potential detrimental effects of ICIs and it was characterised by the increased rate of tumour growth with respect to radiological imaging performed before the start of immunotherapy [7–10]. Retrospective analyses have confirmed that the two phenomena are not fully overlapping [11] and different biological mechanisms at their basis could be hypothesised.

Here, we aim to characterise patients experiencing HPD and early death (ED) following ICIs administration using liquid biopsy to quantify cfDNA and to screen for genetic alterations at baseline and at an early timepoint after treatment.

## Patients and methods

### Patients and plasma sample collection

According to the spontaneous prospective study called MAGIC-1 approved by the Istituto Oncologico Veneto Ethics Committee (protocol number 2016/82, 12/12/2016), we prospectively enrolled all advanced *EGFR-ALK-ROS1* wild-type NSCLC patients starting systemic treatment at our Institution between January 2017 and August 2019 [12]. Eligibility criteria were: availability of tumour biopsy material collected before starting any treatment, the planning of the systemic treatment and the possibility of an adequate clinical and radiological follow-up. Patients were treated according to clinical practice with chemotherapy or ICIs and palliative local treatment was allowed according to the treating physician’s choice.

As previously described [12], liquid biopsy samples were collected at pre-specified timepoints during treatment: at the time of first administration of systemic treatment (baseline, T1), after 3 or 4 weeks of treatment (according to the treatment schedule) (3 ± 1 w, T2), at first radiological restaging (T3), and at radiological progression (PD, T4).

Written informed consent was obtained from all patients before study entry. The study was conducted in accordance with the precepts of the Helsinki declaration.

For this study, only patients receiving single-agent ICIs were considered, and molecular analyses were performed in plasma samples collected at T1 and T2.

Patients experiencing ED were defined as patients experiencing death related to lung cancer within 12 weeks from the start of ICI [6].

Patients having at least two computed tomography (CT) scans available before the start of ICI were evaluated for the presence of HPD. Baseline CT scan was performed within 6 weeks before the start of ICI, and a minimum of 3 weeks between the two previous CT scans were required. Radiological imaging was evaluated by using RECIST v1.1 criteria. Tumour growth rate (TGR) was defined according to previously published criteria [13, 14] and progressive disease (PD) was defined as HPD when TGR measured during ICI exceeds 50% TGR measured before ICI [10].

Among patients not experiencing HPD or ED, we analysed as control group patients experiencing PD not matching HPD criteria and patients deriving clinical benefit (CB) from ICIs, when plasma DNA available was suitable for NGS analysis (Supplemental Fig. 1). CB was defined as no evidence of PD within 6 months since the beginning of ICIs.

### Plasma sample collection

At each timepoint, blood samples (~20 ml) were collected in two cell-free DNA BCT tubes (Streck Corporate, La Vista, NE, USA) and processed within 24–72 h. Plasma was collected as previously described (11). Briefly, blood samples were centrifuged at 2000 × *g* for 10 min at 4 °C, and next, the supernatant was centrifuged at 20,000 × *g* for 10 min. Plasma was stored at −80 °C, until its use.

### cfDNA extraction and quantity and quality assessment

Molecular analyses were performed on patients complying clinical inclusion criteria and having adequate plasma DNA available.

cfDNA was extracted from 2 to 5 mL of plasma using the AVENIO cfDNA Isolation Kit (Roche Diagnostics Spa, Monza, Italia) and eluted into 60 μL of Elution Buffer, according to the manufacturer’s instructions. cfDNA was quantified using the QuBit dsDNA HS Assay kit with QuBit 3.0 fluorimeter (Thermo Fisher Scientific, San Jose, CA), and cfDNA quality was assessed by Agilent Bioanalyzer using a High Sensitivity kit (Agilent Technologies, Palo Alto, CA). The extracted cfDNA was stored at −20 °C until analysis.

### cfDNA sequencing

Sequencing libraries were prepared from 10 to 50 ng cfDNA, using the AVENIO ctDNA Expanded kit (77 genes; Roche Diagnostics Spa), according to the manufacturer’s instructions, and as previously described [15]. Individual enriched libraries were quantified with the QuBit dsDNA HS Assay kit (Thermo Fisher Scientific), and their profile was assessed using the Agilent High Sensitivity kit on the Agilent 2100 Bioanalyzer.

Eight purified libraries per run were pooled and sequenced on an Illumina NextSeq 500 (Illumina, Inc.), using the 300-cycle NextSeq High Output kit, in paired-end mode (2 × 151 cycles).

Analysis and variant calling was performed using the AVENIO ctDNA analysis software (Roche Diagnostics), with default parameter settings for the Expanded Panel.

Only variants with a variant allele fraction (VAF) ≥0.5% and annotated as pathogenic, likely pathogenic or with uncertain significance were taken into account as trackable mutations in plasma samples.

### Statistical analysis

NGS results were elaborated by considering as “non informative” all cases with no genetic alterations detected in plasma samples both at T1 and at T2, which were not included for statistical analysis. To analyse the impact of genetic alterations in plasma on outcome endpoints, in the presence of multiple mutations, the one with the highest VAF (maxVAF) at baseline was considered as the reference, and its value was considered a continuous variable. Statistical analyses were performed also considering the mean VAF as the reference, and we observed a full concordance with analyses by considering the mean values of VAF instead of maxVAF (data not shown). For quantitative evaluation, VAF data below the LOD (0.5% VAF, as previously assessed [15]) at a single timepoint were replaced with a random number from a uniform distribution on the interval [LOD/2, LOD].

Quantification of cfDNA was considered as continuous variable. Quantitative variables were summarised as median and interquartile range (IQ), categorical variables as counts and percentages. The distribution of cfDNA and maxVAF among clinical variables was verified using the Kruskal–Wallis test and pairwise comparisons used the Wilcoxon rank-sum exact test. The correlation between molecular variables has been tested by using the Spearman test with a *P* value < 0.05 considered as significant.

The impact of clinical predictors on the probability of experiencing HPD or ED was estimated in univariate and multiple logistic regression models. Further, the association of cfDNA and maxVAF with HPD or ED was evaluated in separate logistic regression models, adjusted with clinical factors found significant at multiple analysis. Each biomarker was also considered as a categorical variable according to high and low levels. Optimal cut-points were selected in the full sample using a criterion based on maximising the Youden index, being the difference between true positive rate and false positive rate over all possible cut-point values, and validated with bootstrapping. The odds ratios (OR) were reported with their 95% confidence interval (CI). The median follow-up time was based on the reverse Kaplan–Meier estimator.

Radiological response (RR) was assessed by using RECIST criteria v1.1. For the current analysis, CB was defined as stable disease (SD) plus partial response (PR) plus complete response as best RR. Progression-free survival (PFS) was calculated as the time from the beginning of the systemic treatment (corresponding to T1- the time of the baseline sample draw) to radiological PD or death for any cause. Overall survival (OS) was calculated as the time from the beginning of the systemic treatment to death from any cause. Patients who did not develop an event during the study period were censored at the date of the last observation. Median PFS and OS were estimated using the Kaplan–Meier method and reported with their 95% CI calculated according to Brookmeyer and Crowley.

All statistical tests used a two-sided 5% significance level and a *P* value <0.05 was considered statistically significant. Statistical analyses were performed using the SAS statistical package (SAS, rel. 9.4; SAS Institute Inc.), RStudio (RStudio: Integrated Development for R. RStudio, Inc., Boston, MA),) and the cutpointr package of R software.

## Results

### Study population, treatments and outcome

A total of 171 aNSCLC patients enrolled in the MAGIC-1 study and receiving ICIs were evaluated for the current study. Details about clinical features of the whole population and treatments received are summarised in Table 1.

**Table 1:** Clinical features of patients treated with ICIs.

		Study population (*N* = 171)	Molecular subset (*N* = 32)
Age at diagnosis	Median (Q1, Q3)	67 (62, 73.5)	65.5 (60.5, 71)
Sex	0	109 (63.7%)	24 (75.0%)
	1	62 (36.3%)	8 (25.0%)
Smoking	No	25 (14.6%)	2 (6.2%)
	Yes	56 (32.7%)	10 (31.2%)
	Former	90 (52.6%)	20 (62.5%)
Performance status	0	76 (44.4%)	15 (46.9%)
	1	90 (52.6%)	15 (46.9%)
	2	5 (2.9%)	2 (6.2%)
Histology	Adenocarcinoma	132 (77.2%)	25 (78.1%)
	Squamous	26 (15.2%)	6 (18.8%)
	Other	13 (7.6%)	1 (3.1%)
PD-L1	N-Miss	48	7
	Negative	40 (32.5%)	15 (60.0%)
	Positive	83 (67.5%)	10 (40.0%)
PD-L1	N-Miss	48	7
	<50%	59 (48.0%)	17 (68.0%)
	≥50%	64 (52.0%)	8 (32.0%)
Extrathoracic sites	0	67 (39.2%)	13 (40.6%)
	1	50 (29.2%)	8 (25.0%)
	>1	54 (31.6%)	11 (34.4%)
Number of metastatic sites	0–1	80 (46.8%)	18 (56.2%)
	2–4	91 (53.2%)	14 (43.8%)
Treatment lines	1	67 (39.2%)	9 (28.1%)
	>1	104 (60.8%)	23 (71.9%)

Clinical characteristics of patients of the whole treated population with ICIs and of patients considered for molecular evaluation.

Median follow-up was 21.6 (95% CI: 16.8–24.5) months. Median overall survival (OS) was 12.1 (95% CI: 8.2–13.4) months. Median PFS was 5.8 (95% CI: 4.6–6.8) months.

Five patients (3%) experienced progression matching radiological criteria for HPD, 31 patients experienced ED, and one of them met also radiological HPD criteria (Supplemental Fig. 1).

In order to test the hypothesis that longitudinal liquid biopsy could be able to identify patients at higher risk for dismal outcome or detrimental effects, we considered all patients experiencing HPD and ED with plasma DNA suitable for NGS analyses both at baseline and at the earliest timepoint during treatment (T2). All HPD cases and 12 out of 31 ED cases fit this criterion and were analysed to test the impact of molecular variables on outcome (Supplemental Fig. 1). For statistical analysis, the patient experiencing ED and also matching radiological criteria for HPD was considered in the group of HPD patients. Control patients not experiencing HPD or ED included 16 cases (Supplemental Fig. 1).

Clinical features of analysed patients are summarised in Table 1 and are not significantly different from those of the whole study population (data not shown).

### HPD and ED: clinical features and outcome

Among HPD-ED patients (*N* = 35), 26 (74%) had PD-L1 status evaluated and 11 (42%) had level of expression >/= 50%. Among HPD-ED patients, 10 ED cases received first-line pembrolizumab. The other ED cases received immunotherapy after failure of one (*N* = 16) ore two lines (*N* = 5) of chemotherapy and, among them ten received nivolumab, nine atezolizumb and two pembrolizumab. Patients experiencing HPD were treated with nivolumab (*N* = 4) or atezolizumab after the failure of one (*N* = 4) or two chemotherapy regimens (*N* = 1). Median OS of HPD patients was 3.8 (95% CI: 1.7—N.A) months versus 12.4 (95% CI: 9–13.7) months of non-HPD patients (*P* = 0.012) (Supplemental Fig. 2).

We tested the hypothesis that clinical features might be associated with increased probability of experiencing HPD and/or ED. Logistic regression described in Supplemental Table 1 showed that the presence of more than one extrathoracic metastatic site was associated with higher risk of experiencing ED and/or HPD (*P* = 0.002).

### Molecular analysis of longitudinal liquid biopsy

Circulating free DNA (cfDNA) from plasma samples collected at T1 and T2 of 32 patients was analysed, for a total of 64 samples (Supplemental Fig. 1 and Supplemental Table 2).

cfDNA concentrations assessed at baseline (T1) ranged from 3.97 ng per ml of plasma to 290.36 ng per ml of plasma, with a median value of 13.34 ng per ml of plasma (Supplemental Table 2).

All cfDNA samples were found to be adequate for the subsequent NGS analysis, in term of quality and quantity. Sequencing parameters of all analysed samples are reported in Supplemental Table 3. All analysed samples showed a theoretical sensitivity at unique depth greater than 99%, thus enabling a limit of variant detection (LOD) of 0.5%.

At baseline, nine patients were negative for the detection of genetic alterations; among them, at least one alteration was detected at T2 in two patients. As mentioned in “Materials and methods”, we excluded from the analysis all samples found negative at the two consecutive timepoints.

maxVAF quantification was correlated with cfDNA quantification at each timepoint (rho = 0.59 (95% CI: 0.25–0.8), *P* = 0.006 for T1 and rho = 0.58 (95% CI: 0.23–0.8) for T2), *P* = 0.006, but it was interesting to note as the relative variation of maxVAF (T2–T1/T1) was found independent from quantification of cfDNA either at T1 (rho = −0.16 (95% CI: −0.53–0.26), *P* = 0.51) or at T2 (rho = 0.18 (95% CI: −0.24–0.55), *P* = 0.50) (Supplemental Table 4).

### cfDNA concentration identification of ED and HPD patients

cfDNA concentration at any timepoint was not significantly correlated with clinical features (Supplemental Table 5).

**Fig. 1:**
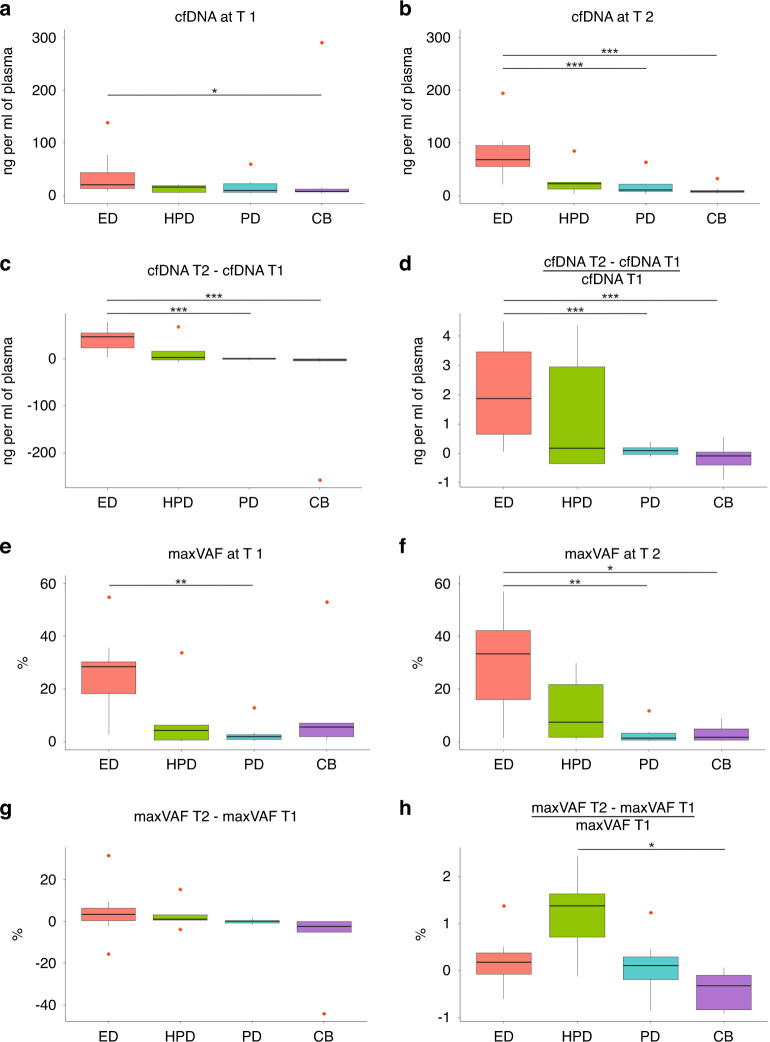
Longitudinal liquid biopsy according to clinical outcome. Box plots show the cfDNA concentration (ng per ml of plasma) or the maxVAF (the highest variant allele fraction, %) at the different timepoints (T1, T2) or their variation in the four clinically defined subgroups of patients: hyperprogressive patients (HPD), patients who died within 12 weeks since the start of ICIs (ED), patients experiencing radiological progressive disease not matching criteria for HPD and patients not progressing within 6 months (CB). *P* values are reported in figure: *P < 0.05; **P < 0.01; ***P < 0.001. **a** cfDNA concentration at T1 (cfDNA1). **b** cfDNA concentration at T2 (cfDNA2). **c** cfDNA absolute variation T1–T2 (cfDNA2-1). **d** cfDNA relative variation T1–T2 [(cfDNA2-cfDNA1)/cfDNA1]. **e** maxVAF at T1. **f** maxVAF at T2. **g** maxVAF absolute variation (VAF2-1). **h** maxVAF relative variation T1–T2 [(VAF2-VAF1)/VAF1].

We investigated the role of cfDNA concentration at different timepoints in predicting ED or HPD. While baseline cfDNA concentration was not associated with the risk of experiencing either ED or HPD (Table 2A, B), a significant difference in the median concentrations of cfDNA at T2 and in its variation during treatment were observed among the four clinically defined subgroups of patients (HPD, ED, CB versus PD) (*P* < 0.001 for T2, *P* < 0.001 for the absolute difference and *P* = 0.002 for the relative change from T1 to T2) (Fig. 1b–d, and Table 3A).

**Table 2:** Logistic regression predicting the risk of experiencing ED or HPD according to cfDNA concentration or to VAF.

(A)	Univariate analysis		Multivariate analysis	
	OR (95% CI)	*P* value	Adjusted OR *(95% CI)	*P* value
cfDNA T1	1.00 (0.99, 1.02)	*0.608*	1.00 (0.99, 1.02)	*0.553*
cfDNA T2	1.06 (1.02, 1.1)	*0.002*	1.06 (1.02, 1.16)	***0.012***
cfDNA T2-cfDNA T1	1.08 (1.03, 1.13)	*0.003*	1.08 (1.02, 1.14)	***0.005***
${\frac{\mbox{cfDNA T2-cfDNA T1}}{{\mbox{cfDNA T1}}}}$	2.35 (1.24, 4.44)	*0.009*	3.08 (1.32, 7.2)	***0.009***

(B)	Univariate analysis	
	OR (95% CI)	*P* value
cfDNA T1	0.97 (0.9, 1.05)	*0.48*
cfDNA T2	0.99 (0.96, 1.02)	*0.604*
cfDNA T2-cfDNA T1	1.0 (0.98, 1.03)	*0.734*
${\frac{\mbox{cfDNA T2-cfDNA T1}}{{\mbox{cfDNA T1}}}}$	1.24 (0.7, 2.2)	*0.467*

(C)	Univariate analysis		Multivariate analysis	
	OR (95% CI)	*P* value	Adjusted OR *(95% CI)	*P* value
maxVAF T1	1.07 (1.01, 1.14)	*0.029*	1.08 (1.01, 1.16)	***0.029***
maxVAF T2	1.11 (1.03, 1.21)	*0.01*	1.13 (1.02, 1.24)	***0.017***
maxVAF T2- maxVAF T1	1.07 (0.96, 1.19)	*0.229*	1.07 (0.96, 1.19)	*0.225*
${\frac{\mbox{maxVAF T2- maxVAF T1}}{{\mbox{maxVAF T1}}}}$	0.89 (0.31, 2.55)	*0.827*	0.94 (0.31, 2.82)	*0.915*

(D)	Univariate analysis	
	OR (95% CI)	*P value*
maxVAF T1	0.98 (0.91, 1.05)	*0.5*
maxVAF T2	0.99 (0.94, 1.05)	*0.845*
maxVAF T2- maxVAF T1	1.03 (0.94, 1.14)	*0.505*
${\frac{\mbox{maxVAF T2- maxVAF T1}}{{\mbox{maxVAF T1}}}}$	8.14 (1.38, 47.96)	***0.02***

Logistic regression predicting risk of experiencing early death (ED) or hyperprogression (HPD) according to the concentration of cfDNA or according to the value of the highest VAF (maxVAF) at baseline among all the individual genetic alteration, at T1, at T2, or its absolute (T2–T1) or relative variation T1–T2 (T2–T1/T1).

^*^HR adjusted for extrathoracic sites.

(A) Logistic regression predicting the risk of experiencing ED according to cfDNA concentration, evaluated as a continuous variable (*n* = 11 ED patients/32 analysed patients).

(B) Logistic regression predicting the risk of experiencing HPD according to cfDNA concentration, evaluated as a continuous variable (*n* = 5 HPD patients/32 analysed patients).

(C) Logistic regression predicting the risk of experiencing ED according to maxVAF, evaluated as a continuous variable (*n* = 8 ED patients/25 analysed patients).

(D) Logistic regression predicting the risk of experiencing HPD according to VAF (%), evaluated as a continuous variable (*n* = 5 HPD patients/25 analysed patients).

Statistically significant *P* < 0.05 values are in bold italic.

Statistically significant *P*-values are in italic.

**Table 3:** Median concentration of cfDNA or maxVAF at T1, T2 and its variation T1–T2 according to clinical outcome.

(A)	ED (*N* = 11)	HPD (*N* = 5)	PD (*N* = 8)	CB (*N* = 8)	Total (*N* = 32)	*P* value
						KW test
cfDNA T1 median (Q1–Q3)	20.25 (13.34, 43.48)	15.81 (5.76, 19.13)	9.36 (5.85, 22.40)	8.20 (7.05, 12.49)	13.34 (7.58, 22.35)	*0.128*
cfDNA2 median (Q1–Q3)	67.82 (55.37, 94.64)	22.75 (12.40, 25.59)	10.80 (7.74, 21.16)	7.44 (6.21, 9.39)	21.94 (8.32, 66.87)	***<0.001***
cfDNA T2- cfDNA T1 median (Q1–Q3)	47.57 (23.42, 55.36)	3.79 (−1.72, 16.99)	1.15 (−0.35, 1.75)	−1.45 (−3.55, 0.32)	2.47 (−0.35, 35.29)	***<0.001***
${\frac{\mbox{cfDNA T2-cfDNA T1}}{{\mbox{cfDNA T1}}}}$ median (Q1–Q3)	1.87 (0.65, 3.45)	0.17 (−0.35, 2.95)	0.09 (−0.04, 0.19)	−0.10 (−0.41, 0.04)	0.15 (−0.04, 1.51)	***0.002***

(B)	ED (*N* = 11)	Other (*N* = 21)	*P* value
			KW test
cfDNA T1 median (Q1–Q3)	20.25 (13.34, 43.48)	8.40 (5.89, 19.13)	***0.018***
cfDNA T2 median (Q1–Q3)	67.82 (55.37, 94.64)	9.07 (6.32, 22.44)	***<0.001***
cfDNA T2- cfDNA T1 median (Q1–Q3)	47.57 (23.42, 55.36)	0.28 (−2.83, 2.32)	***<0.001***
${\frac{\mbox{cfDNA T2-cfDNA T1}}{{\mbox{cfDNA T1}}}}$ median (Q1–Q3)	1.87 (0.65, 3.45)	0.03 (−0.19, 0.17)	***<0.001***

(C)	ED (*N* = 8)	HPD (*N* = 5)	PD (*N* = 7)	CB (*N* = 5)	Total (*N* = 25)	*P* value
						KW test
maxVAF T1 median (Q1–Q3)	28.45 (18.25, 30.30)	4.32 (0.73, 6.32)	1.92 (0.92, 2.70)	5.53 (1.93, 7.13)	4.32 (1.92, 28.39)	***0.046***
maxVAF T2 median (Q1–Q3)	33.46 (16.05, 42.10)	7.39 (1.73, 21.75)	1.41 (0.63, 3.21)	1.74 (0.59, 4.83)	4.06 (1.41, 21.75)	***0.016***
maxVAF T2-maxVAFT1 median (Q1–Q3)	3.38 (0.43, 6.47)	1.00 (0.52, 3.07)	0.07 (−0.83, 0.29)	−2.30 (−5.04, −0.19)	0.16 (−1.63, 2.22)	*0.062*
${\frac{\mbox{maxVAF T2-maxVAFT1}}{{\mbox{maxVAF T1}}}}$ median (Q1–Q3)	0.18 (−0.08, 0.37)	1.37 (0.71, 1.62)	0.10 (−0.19, 0.29)	−0.32 (−0.83, −0.10)	0.10 (−0.30, 0.50)	***0.033***

(D)	ED (*N* = 8)	Other (*N* = 17)	*P* value
			KW test
maxVAF T1 median (Q1–Q3)	28.45 (18.25, 30.30)	2.00 (0.73, 6.32)	***0.01***
maxVAF T2 median (Q1–Q3)	33.46 (16.05, 42.10)	1.74 (0.75, 7.39)	***0.004***
maxVAF T2-maxVAF T1 median (Q1–Q3)	3.38 (0.43, 6.47)	0.03 (−1.63, 0.52)	*0.081*
${\frac{\mbox{maxVAF T2-maxVAF T1}}{{\mbox{maxVAF T1}}}}$ median (Q1–Q3)	0.18 (−0.08, 0.37)	0.05 (−0.30, 0.71)	*0.727*

(E)	HPD (*N* = 5)	Other (*N* = 20)	*P* value
			KW test
maxVAF T1 median (Q1–Q3)	4.32 (0.73, 6.32)	4.72 (1.93, 28.42)	*0.541*
maxVAF T2 median (Q1–Q3)	7.39 (1.73, 21.75)	3.94 (1.24, 23.27)	*0.786*
maxVAF T2-maxVAF T1 median (Q1–Q3)	1.00 (0.52, 3.07)	0.05 (−1.80, 1.64)	*0.342*
${\frac{\mbox{maxVAF T2-maxVAF T1}}{{\mbox{maxVAF T1}}}}$ median (Q1–Q3)	1.37 (0.71, 1.62)	0.05 (−0.35, 0.23)	***0.021***

cfDNA concentration—ng per ml of plasma.

(A) Median concentrations of cfDNA (ng per ml of plasma) and interquartile range (Q1–Q3) in the four clinically defined subgroups of patients (HPD, ED, CB versus PD).

(B) Median concentration of cfDNA (ng per ml of plasma) and interquartile rage (Q1–Q3) in the ED subgroup and in the other patients not experiencing ED.

(C) Median value of the maxVAF (%) and interquartile rage (Q1–Q3) in the four clinically defined subgroups of patients (HPD, ED, CB versus PD).

(D) Median value of the maxVAF (%) and interquartile rage (Q1–Q3) in the ED subgroup and in the other patients not experiencing ED.

(E) Median value of the maxVAF (%) in the HPD subgroup and in the other patients not experiencing HPD.

Values are median and interquartile range (Q1-Q3).

Statistically significant *P* < 0.05 values are in bold italic.

Statistically significant *P*-values are in italic.

Specifically, median concentration of cfDNA at T2 was 67.82 (ng per ml of plasma) (95% CI: 55.37–94.64) in the ED subgroup, versus 9.07 (95% CI: 6.32–22.44) for patients not experiencing ED (*P* < 0.001) (Table 3B). A greater variation in cfDNA concentration during treatment in ED patients was also shown (*P* < 0.001 for both the absolute and relative difference from T1 to T2) (Table 3A and Supplemental Fig. 3). Interestingly, logistic regression confirmed that both T2 concentration and variation T1–T2 permit to identify the risk of experiencing ED (Table 2A), also when analysing the impact of clinical factors affecting the risk for ED (Supplemental Table 1).

On the other hand, neither cfDNA concentration at T2 nor its variation T1–T2 were associated with the risk of HPD (Table 2B).

### Monitoring of plasma genotyping and identification of ED and HPD patients

At least one somatic variation was identified in 72% (23/32) of plasma samples at baseline, with an average of two mutations per sample. The most frequent mutations were found in *TP53* (28%), *KRAS* (10%), *APC* (5%) and *STK11* (5%) genes (Supplemental Table 2). VAF of individual detected genetic alterations ranged from 0.5 to 52.88%, with a median of 4.71% (Supplemental Table 2).

We first evaluated the potential impact of clinicopathological features on the parameter maxVAF, used as a reference value for NGS results, but no correlation was found between maxVAF at any timepoint and clinical features (Supplemental Table 6).

When we tested potential impact of maxVAF on outcome endpoints, we observed that the maxVAF value detected at baseline and at T2, considered as a static parameter, was statistically associated with an increased risk of experiencing ED (Table 2C). In particular, the median value of maxVAF at T2 was 33.46 (95% CI: 16.05–42.10) for patients experiencing ED versus 1.74 (95% CI: 0.75–7.39) for the rest of the study population (*P* = 0.004; Table 3D, Fig. 1).

Moreover, a significant difference in the median of the relative variation (T2–T1)/T1 among the four clinically defined subgroups of patients (*P* = 0.033, Table 3C, Fig. 1h) was observed. Specifically, the median (T2–T1)/T1 variation was statistically higher in the HPD patients compared with the other patients (1.37, (95% CI: 0.71–1.62) versus 0.05 (95% CI: −0.35–0.23, *P* = 0.021), (Table 3E). Logistic regression confirmed that the relative increase in maxVAF from T1 to T2 was able to identify the risk of experiencing HPD (OR = 8.14, 95% CI: 1.38–47.96, *P*  = 0.02) (Table 4D).

**Table 4:** Logistic regression predicting the risk of experiencing ED or HPD according to cfDNA concentration or VAF as a categorical variable.

(A)		Univariate analysis		Multivariate analysis	
		OR (95% CI)	*P* value	Adjusted OR* (95% CI)	*P* value
cfDNA T1	High vs low (12.15)	4.84 (1.09, 21.58)	*0.039*	4.42 (0.92,21.2)	*0.063*
cfDNA T2	High vs low (22.75)	21 (3.26,135.48)	***0.001***	22.14 (2.95, 166.34)	***0.003***
cfDNA T2-cfDNA T1	High vs low (3.79)	65 (6,703.67)	***0.001***	68.18 (5.61, 828.57)	***0.001***
${\frac{\mbox{cfDNA T2-cfDNA T1}}{{\mbox{cfDNA T1}}}}$	High vs low (0.17)	13 (2.4, 70.46)	***0.003***	17.21 (2.58, 114.67)	***0.003***

(B)		Univariate analysis	
		OR (95% CI)	*P* value
maxVAF T1	High vs low (4.32)	0.67 (0.09,4.89)	*0.69*
maxVAF T2	High vs low (0.84)	1.33 (0.12,14.9)	*0.815*
maxVAF T2- maxVAF T1	High vs low (0.52)	2.79 (0.37,20.82)	*0.318*
${\frac{\mbox{maxVAF T2- maxVAF T1}}{{\mbox{maxVAF T1}}}}$	High vs low (0.71)	13.5 (1.34, 135.97)	***0.027***

*HR adjusted for extrathoracic sites.

(A) Logistic regression predicting the risk of experiencing ED or HPD according to cfDNA concentration (ng per ml of plasma) as a categorical variable (high versus low) (*n* = 16 experiencing ED or HPD/32 analysed patients).

(B) Logistic regression predicting the risk of experiencing HPD according to maxVAF (%) as a categorical variable (high versus low) (*n* = 5 experiencing HPD /25 analysed patients).

Statistically significant *P* < 0.05 values are in bold italic.

Statistically significant *P*-values are in italic.

### Cut-off definition and proposal for HPD/ED risk assessment in clinical practice

In order to investigate the potential applicability of our results, we defined the optimal cut-off value for cfDNA levels and maxVAF able to individuate patients at higher risk to develop dismal outcome or detrimental effects following ICIs treatment.

Through a ROC-based analysis, we determined the value of 22.7 ng per ml of plasma as the optimal cut-off of the cfDNA concentration at T2 (corresponding to the lower quartile limit) to discriminate between patients experiencing ED or HPD versus all other patients, with an accuracy of 81% (95% CI: 64–93). The median cut-point value in the boostrap samples was 22.7 (95% CI: 12.4–67.1), with an accuracy of 88% (95% CI: 78–94). Patients with a cfDNA concentration above this value presented an increased risk of experiencing potential either HPD or ED effect with an adjusted OR of 22.1 (95% CI: 2.9–166.3, *P*  = 0.003) (Table 4A).

Similarly, we identified the optimal cut-off of cfDNA absolute and relative variation from T1 to T2. An absolute change of 3.8 ng per ml of plasma or a relative increase of 0.2 ng per ml of plasma (T1–T2) identified an increased risk of ED or HPD with an adjusted OR of 68.2 (95% CI: 5.6–828.6, *P* = 0.001) and 17.2 (95% CI: 2.6–114.7, *P* = 0.003), respectively (Table 4A). The performance of optimal cutpoins is reported in Supplemental Table 7.

Since cfDNA quantification was not able to specifically identify all HPD patients, we analysed the maxVAF to define a cut-off value for the risk of experiencing HPD: the value of 0.71 for maxVAF relative increase T1–T2 emerged as the optimal cut-off, with an accuracy of 84% (95% CI: 64–95). Specifically, patients with maxVAF relative variation T2–T1 exceeding this value had an increased risk for experiencing HPD, with an OR of 13.5 (95% CI: 1.3–136.0, *P*  = 0.027) (Table 4B).

## Discussion

The introduction of ICIs in clinical practice has radically changed the outcome of non-oncogene addicted aNSCLC patients [16, 17]. Even though ICIs are associated with the chance of long survivorship, their benefit is highly heterogeneous and some detrimental effects have been described [18–20]. Importantly, ED and HPD have been observed even in aNSCLC patients expressing high levels of PD-L1 and treated in first-line with single-agent or combination ICIs [3, 21] while no biomarkers are currently available for their identification.

In our report, we assessed the potential value of liquid biopsy at early timepoints during ICIs treatment for assessing the risk for potential detrimental effects. HPD is a phenomenon related to largely unknown biological mechanisms triggered by ICIs leading to accelerated tumour growth. HPD is complex to assess in clinical practice [7, 8] and no unique defining criteria are available [10, 22] although the need for taking into consideration both clinical and radiological criteria has already been raised [9]. Among potential clinical criteria, we decided to include patients experiencing death within 12 weeks from the start of ICIs, being an objective criterion and a phenomenon already observed in several clinical trials [6, 11]. Evidence about lack of complete overlap between the two phenomena, ED and HPD, in line with previous observations [23, 24], has been confirmed in our experience. We also confirmed the potential impact of extrathoracic disease on the risk of developing ED or HPD and the occurrence of ED even in patients expressing high level of PD-L1 and treated in a first-line setting [3, 10, 25].

When considering the impact of longitudinal liquid biopsy, we analysed both cfDNA values and VAF of tumour-associated genetic alterations during treatment: our results permit to speculate a differential role of the two assessed parameters and a potential different biological background for the ED and HPD phenomena. Specifically, ED was associated with a dramatic variation in cfDNA concentration between T1 and T2, but no significant change of maxVAF between T1 and T2. Although it is generally hold that ctDNA represents only a small portion of total cfDNA [26], we speculate that in the ED subgroup ctDNA might represent a large part of cfDNA. Although the NGS assay used in this study did not enable estimation of the tumour fraction in cfDNA, our hypothesis is supported by the much higher maxVAF value of tumour-associated mutations in ED samples both at T1 and T2, compared with other samples (Table 3C, D). Expectedly, we did not observe a variation in the maxVAF value between T1 and T2, likely due to the fact that this parameter reached a plateau value in ED patients. On the other hand, cfDNA concentration was not associated with HPD, whereas the dynamic relative variation of maxVAF T1–T2 identified patients experiencing HPD. Since maxVAF relative variation is independent from the cfDNA, it might be more influenced by rapid increase in tumour growth following the start of ICIs and less related to baseline prognostic factors.

The role of liquid biopsy in patients with solid tumours and its predictive potential on outcome has already been described [12, 27–29], but identification of potential detrimental effects of ICIs requires early time-point evaluation. To the best of our knowledge, our study is the first evaluating the impact of liquid biopsy performed after 3–4 weeks of treatment [12] and the first assessment concerning the identification of HPD and ED by using liquid biopsy. cfDNA quantification is easy to perform and could be assessed at low cost in clinical practice. We thus suggest a two-step risk assessment model, including an initial evaluation of cfDNA in plasma at T1 and T2 followed by NGS (Supplemental Fig. 4). Although limited by the relatively low number of patients included, this approach represents a proof-of-concept analysis in order to show potential clinical applicability of our longitudinal liquid biopsy model.

Early identification of ED/HPD patients has great potential for clinical applications as it could help optimisation and personalisation of treatment, thus avoiding more toxic combination treatment when not needed. In view of the main limitation of our study, represented by the relatively small number of cases analysed, the prospective interventional trial is warranted to confirm our results and validate a dynamic risk-based treatment approach.

In conclusion, this study represents a proof-of-concept analysis concerning an innovative approach to the issue of predictive biomarkers of immunotherapy in lung cancer, focusing on patients who do not derive any clinical benefit and could benefit of a customised approach including early changes in treatment.

## Supplementary information

Supplemental materials

Supplemental Figure 1

Supplemental Figure 2

Supplemental Figure 3

Supplemental Figure 4

aj checklist

## Data Availability

The data generated and analysed during this study are included in this published article and its additional files. Further raw data might be asked to the authors.
