# Supplementary material for: Longitudinal liquid biopsy anticipates hyperprogression and early death in advanced non-small cell lung cancer patients treated with immune checkpoint inhibitors
Source: Br J Cancer. 2022 Sep 29;127(11):2034–42. doi: 10.1038/s41416-022-01978-1 (PMC9681746; doi:10.1038/s41416-022-01978-1)
Supplement: Supplementary file 1 — Supplemental materials [file 41416_2022_1978_MOESM1_ESM.docx]

**SUPPLEMENTAL MATERIAL.**

**SUPPLEMENTAL FIGURE LEGENDS.**

**Supplemental Figure 1.**

Flow chart of study population. Radiological response was assessed by RECIST criteria v1.1. (PD: Progressive Disease; SD: stable disease; PR: Partial Response). NGS feasibility for control group was assessed among patients complying with clinical criteria as referred in method section.

**Supplemental Figure 2. Overall survival in patients experiencing and not experiencing hyperprogression.**

Kaplan-Meier curves showing overall survival (OS) of the overall study population not experiencing Hyperprogression (HPD) and of patients experiencing HPD. The hazard ratios with 95% confidence interval and p-values are reported in figure.

**Supplemental Figure 3. Variation from T1 to T2 in the cfDNA concentration during treatment according to clinical outcome.**

Variation in the cfDNA concentration (ng per ml of plasma) observed from baseline to T2 in each analysed patient (A), and according to clinical response (ED, HPD, PD and CB) (B).

**Supplemental Figure 4. Flow chart for early identification of detrimental effects during ICIs treatment of NSCLC patients.**

We propose a two-step risk assessment model for early identification of ED and HPD patients during immunotherapy. We propose to perform liquid biopsy at baseline (T1) and after three or four weeks of treatment (T2). Quantification of cfDNA enables to identify patients at higher risk of ED and partially patients at higher risk of HPD. Patients presenting cfDNA at T2 above the pre-established cut-off values are considered at high-risk of detrimental effects and could be discussed for treatment modification. In the presence of cfDNA levels below the cut-off value, NGS analysis is suggested in order to identify patients at higher risk for HPD based on VAF values. Cut-off values indicated in the figure are those identified in this study.

**SUPPLEMENTAL TABLES.**

**Supplementary Table 1. Logistic regression predicting the risk of experiencing early death and/ or hyperprogression according to clinical characteristics.**

|  |  | **detrimental effects/N** | **univariate** | | **multiple** | |
| --- | --- | --- | --- | --- | --- | --- |
|  |  |  | **OR(95%CI)** | ***P value*** | **OR(95%CI)** | ***P (Wald's test)*** |
| **Age** | continous variable |  | 0.98 (0.94,1.02) | *0.247* |  |  |
| **Sex** | Male | 24/109 | Ref |  |  |  |
|  | Female | 11/62 | 0.76 (0.35,1.69) | *0.506* |  |  |
| **Smoking** | No | 5/25 | Ref |  |  |  |
|  | Yes | 11/56 | 0.98 (0.3,3.19) | *0.97* |  |  |
|  | Former | 19/90 | 1.07 (0.36,3.23) | *0.904* |  |  |
| **Performance status** | 0 | 14/76 | Ref |  |  |  |
|  | 1 | 20/90 | 1.27 (0.59,2.72) | *0.546* |  |  |
|  | 2 | 1/5 | 1.11 (0.11, 10.68) | *0.93* |  |  |
| **Histology** | Adenocarcinoma | 25/132 | Ref |  |  |  |
|  | Squamous | 7/26 | 1.58 (0.6,4.16) | *0.357* |  |  |
|  | Other | 3/13 | 1.28 (0.33,5.01) | *0.719* |  |  |
| PD-L1 | <50% | 15/59 | Ref |  |  |  |
|  | ≥50% | 11/64 | 0.61 (0.25,1.46) | *0.266* |  |  |
| **Stage** | **I-II** | 2/13 | Ref |  |  |  |
|  | III-IV | 33/158 | 1.45 (0.31,6.87) | *0.638* |  |  |
| **Extrathoracic sites** | No | 7/67 | Ref |  | Ref |  |
|  | 1 | 8/50 | 1.63 (0.55,4.85) | *0.377* | 2.26 (0.7,7.24) |  |
|  | >1 | 20/54 | 5.04 (1.93,13.14) | ***< 0.001*** | 12.47 (2.46,63.27) | *0.002* |
| **N.r of metastatic sites** | 0-1 | 12/80 | Ref |  |  |  |
|  | 2-4 | 23/91 | 1.92 (0.88,4.16) | *0.1* |  |  |
| **Bone/Liver/Brain** | No | 12/98 | Ref |  |  |  |
|  | Brain | 7/16 | 5.57 (1.75,17.74) | ***0.004*** |  |  |
|  | Liver | 3/11 | 2.69 (0.63,11.55) | *0.184* |  |  |
|  | Bone | 6/27 | 2.05 (0.69,6.09) | *0.197* |  |  |
|  | Multiple | 7/19 | 4.18 (1.38,12.69) | ***0.012*** |  |  |
| **Treatment lines** | 1 | 14/67 | Ref |  |  |  |
|  | >1 | 21/104 | 0.96 (0.45,2.05) | *0.911* |  |  |

**Supplemental Table 2. Details of cfDNA concentration and the single nucleotide variations (SNV) identified at T1 and T2.**

|  |  |  |  | **T1** | | **T2** | |
| --- | --- | --- | --- | --- | --- | --- | --- |
| **Patient ID** | **Clinical features** | **Gene** | **SNV** | **VAF-1 (%)** | **cfDNA concentration^#^** | **VAF-2 (%)** | **cfDNA concentration^#^** |
| #M43 | ED - HPD | *KRAS* | p.Gly12Asp | 4.32 | 5.76 | 7.39 | 22.75 |
|  |  | *KDR* | p.Pro667His | 0 |  | 1.03 |  |
| #M191 | ED | *PMS2* | p.Ala38Val | 54.8 | 16.10 | 57.02 | 25.40 |
|  |  | *RET* | p.Gly733Asp | 27.42 |  | 34.92 |  |
|  |  | *TP53* | p.Ala159Pro | 26.57 |  | 34.86 |  |
|  |  | *TP53* | p.Leu130His | 2.62 |  | 0.58 |  |
|  |  | *APC* | p.Ser1503* | 28.7 |  | 37.46 |  |
| #M243* | ED | *TP53* | p.Arg181Pro | 28.5 | 14.54 | 34 | 67.17 |
|  |  | *KIT* | p.Tyr95Phe | 18.34 |  | 22.31 |  |
| #M288* | ED | *PIK3CA* | p.Glu545Lys | 3.92 | 12.15 | 1.56 | 66.78 |
| #M324 | ED | *CDKN2A* | p.Asp108Asn | 7.69 | 138.26 | 14.05 | 194.35 |
|  |  | *KRAS* | p.Gly12Cys | 28.53 |  | 37.93 |  |
|  |  | *RB1* | p.Gln471* | 14.69 |  | 20.81 |  |
|  |  | *TP53* | p.Glu171* | 14.62 |  | 21.65 |  |
|  |  | *STK11* | p.Glu293* | 16.63 |  | 25.26 |  |
| #M322 | ED | *AR* | p.Val478Leu | 23.03 | 52.57 | 54.61 | 90.65 |
|  |  | *KDR* | p.Tyr927Asp | 0 |  | 1.43 |  |
|  |  | *PDCD1LG2* | c.816+2T>G | 8.38 |  | 29.36 |  |
| #M301 | ED | *AR* | p.Gln58Leu | 0.62 | 77.7 | 0.5 | 82.18 |
|  |  | *NF2* | p.Glu112* | 1.15 |  | 0.66 |  |
|  |  | *TP53* | p.ARG158Leu | 35.59 |  | 20.05 |  |
|  |  | *BRCA2* | p.Thr2350Ser | 32.33 |  | 17.85 |  |
|  |  | *PIK3R1* | p.Ser628Asn | 17.4 |  | 8.52 |  |
|  |  | *RET* | p.Arg79Leu | 12.98 |  | 7.14 |  |
| #M319 | ED | *_* |  |  | 34.38 |  | 98.62 |
| #M215 | ED | *_* |  |  | 20.25 |  | 67.82 |
| #M273 | ED | *_* |  |  | 8.95 |  | 21.43 |
| #M276 | ED | *FBXW7* | p.Arg658* | 2.34 | 9.6 | 5.01 | 43.96 |
|  |  | *TP53* | p.Glu298* | 2.7 |  | 4.06 |  |
| #M300 | ED | *ALK* | p.Pro307Leu | 0.52 | 24 | 0.3 | 103.92 |
|  |  | *IDHI* | p.Arg132His | 7.7 |  | 15.54 |  |
|  |  | *STK11* | c.375-2A>G | 0.67 |  | 0.31 |  |
|  |  | *TP53* | p.Arg249Met | 0.92 |  | 0 |  |
|  |  | *TP53* | p.His168Pro | 28.39 |  | 32.92 |  |
| #M168 | HPD | *PTCH1* | p.Ala793Ser | 0.73 | 4.95 | 1.73 | 3.23 |
| #M182 | HPD | *KRAS* | p.Ala146Thr | 4.75 | 15.81 | 16.53 | 84.80 |
|  |  | *KEAP1* | p.Gly378 | 6.32 |  | 21.75 |  |
| #M202 | HPD | *TP53* | c.993+1G>A | 0 | 21.80 | 0.84 | 25.59 |
| #M257 | HPD | *RB1* | p.Arg262Gln | 15.61 | 19.13 | 12.62 | 12.4 |
|  |  | *TP53* | p.His179Arg | 33.71 |  | 29.84 |  |
|  |  | *KDR* | p.Ser968Ile | 2.72 |  | 2.85 |  |
|  |  | *MSH6* | p.Leu1264Val | 15.64 |  | 12.35 |  |
|  |  | *TERT* | c.-146C>T | 6.5 |  | 0 |  |
| #M148 | PD | *TP53* | p.Gln192* | 2 | 3.97 | 1.41 | 3.73 |
| #M262 | PD | *TP53* | p.Ile255Phe | 0.68 | 7.63 | 0.75 | 8.48 |
| #M204 | PD | *AR* | p.Gln58Leu | 3.39 | 25.44 | 3.82 | 22.44 |
| #M234 | PD | _ |  |  | 21.39 |  | 20.73 |
| #M248 | PD | *KRAS* | p.Gly12Asp | 12.82 | 11.08 | 11.76 | 12.53 |
|  |  | *TP53* | p.Tyr220Cys | 9.31 |  | 8.02 |  |
| #M264 | PD | *KEAP1* | p.Glu134* | 0 | 6.45 | 0.51 | 9.07 |
| #M268 | PD | *APC* | p.Glu1345* | 1.05 | 4.04 | 4.68 | 5.50 |
|  |  | *TP53* | p.Ala159Val | 0 |  | 3.06 |  |
|  |  | *STK11* | p.His154Leu | 1.17 |  | 2.61 |  |
| #M110 | PD | *TP53* | p.Gln331* | 1.92 | 60.00 | 0 | 64.04 |
|  |  | *TP53* | p.Arg248Gln | 0.95 |  | 1.57 |  |
|  |  | *APC* | p.Gln1611Glu | 1.7 |  | 1.68 |  |
| #M185 | CB | *_* |  |  | 15.45 |  | 12.58 |
| #M199 | CB | *_* |  |  | 8.4 |  | 8.33 |
| #M222 | CB | *KDR* | p.Trp364* | 7.13 | 11.50 | 4.83 | 5.90 |
| #M277 | CB | *_* |  |  | 4.28 |  | 6.60 |
| #M284 |  | *AR* | p.Gln58Leu | 1.93 | 8.00 | 1.74 | 8.28 |
|  |  | *TP53* | p.His214Arg | 1.27 |  | 0.92 |  |
| #M238 | CB | *KRAS* | p.Gly12Val | 0.56 | 5.89 | 0.59 | 6.32 |
| #M249 | CB | *BRAF* | p.Gly596Arg | 5.53 | 7.44 | 0 | 4.61 |
|  |  | *TP53* | p.Gly245Val | 3.71 |  | 0 |  |
|  |  | *DDR2* | p.Glu583Lys | 2.26 |  | 0 |  |
|  |  | *KEAP1* | p.Arg536Pro | 4.71 |  | 0 |  |
|  |  | *TSC2* | p.Ser740Cys | 3.94 |  | 0 |  |
| #M251 | CB | *KRAS* | p.Gly12Ala | 52.88 | 290.36 | 8.91 | 32.92 |
|  |  | *TP53* | p.Arg306* | 41.22 |  | 5.79 |  |
|  |  | *MSH2* | p.Arg621Leu | 16.17 |  | 2.89 |  |
|  |  | *MTOR* | p.Ala1792Pro | 0.7 |  | 0 |  |

*EGFR amplification was detected in patient #M243 and a RET-KIFT5 fusion was detected in #M288 patient, both at T1 and T2.

**^#^** cfDNA concentration - ng per ml of plasma

**Supplementary Table 3. Sequencing parameters of all analysed samples.**

Analysis and variant calling was performed using the AVENIO ctDNA analysis software (Roche Diagnostics). The 2sequencing depth” parameter represents the depth of sequenced and aligned on-target reads for the sample, whereas the “unique depth” is the depth of unique sequenced DNA fragments, with duplicates removed. The “theoretical sensitivity” parameter for each sequenced library represents the probability of detecting a variant molecule at a limit of detection (0.5% VAF). This value is calculated for the median, 5th percentile, and 95th percentile of depth within the sample.

| **Patient ID** | **Clinical features** |  | **Input DNA Mass (ng)** | **Sequencing Depth Median** | **Sequencing Depth 5th Percentile** | **Sequencing Depth 95th Percentile** | **Unique Depth Median** | **Unique Depth 5th Percentile** | **Unique Depth 95th Percentile** | **On-Target Rate** | **Theoretical Sensitivity Median** | **Theoretical Sensitivity 5th Percentile** | **Theoretical Sensitivity 95th Percentile** |
| --- | --- | --- | --- | --- | --- | --- | --- | --- | --- | --- | --- | --- | --- |
| #M43 | ED - HPD | **T1** | 21,60 | 16497 | 5278 | 34647 | 4756 | 2093 | 7085 | 74,92% | 100,00% | 99,81% | 100,00% |
|  |  | **T2** | 34,20 | 16350 | 5126 | 33854 | 7518 | 2758 | 12440 | 74,95% | 100,00% | 99,99% | 100,00% |
| #M191 | ED | **T1** | 24,70 | 11659 | 3745 | 27633 | 4829 | 2176 | 8070 | 71,06% | 100,00% | 99,87% | 100,00% |
|  |  | **T2** | 38,90 | 13522 | 4126 | 32588 | 6706 | 2659 | 12209 | 69,68% | 100,00% | 99,98% | 100,00% |
| #M243* | ED | **T1** | 22,30 | 12714 | 3767 | 29362 | 5031 | 2040 | 8406 | 71,55% | 100,00% | 99,77% | 100,00% |
|  |  | **T2** | 50,00 | 12968 | 3136 | 32224 | 7059 | 2159 | 13274 | 72,01% | 100,00% | 99,86% | 100,00% |
| #M288* | ED | **T1** | 46,60 | 15575 | 5184 | 35686 | 6890 | 3053 | 11031 | 75,63% | 100,00% | 100,00% | 100,00% |
|  |  | **T2** | 50,00 | 13750 | 3911 | 30553 | 7243 | 2740 | 11842 | 75,00% | 100,00% | 99,99% | 100,00% |
| #M324 | ED | **T1** | 49,80 | 11454 | 3349 | 25996 | 6486 | 2384 | 10679 | 68,84% | 100,00% | 99,95% | 100,00% |
|  |  | **T2** | 50,00 | 12422 | 3684 | 28098 | 6903 | 2593 | 11407 | 69,28% | 100,00% | 99,98% | 100,00% |
| #M322 | ED | **T1** | 40,30 | 12296 | 3396 | 29583 | 6225 | 2314 | 10516 | 71,64% | 100,00% | 99,93% | 100,00% |
|  |  | **T2** | 50,00 | 13299 | 4233 | 31487 | 6930 | 2881 | 12947 | 71,98% | 100,00% | 99,99% | 100,00% |
| #M301 | ED | **T1** | 51,80 | 10185 | 4086 | 21801 | 5875 | 2793 | 10104 | 71,78% | 100,00% | 99,99% | 100,00% |
|  |  | **T2** | 49,70 | 10648 | 3936 | 22303 | 6404 | 2812 | 10741 | 72,21% | 100,00% | 99,99% | 100,00% |
| #M319 | ED | **T1** | 49,50 | 9480 | 4246 | 17866 | 5452 | 2814 | 8102 | 66,93% | 100,00% | 99,99% | 100,00% |
|  |  | **T2** | 51,30 | 9099 | 3990 | 16235 | 5177 | 2603 | 7574 | 64,12% | 100,00% | 99,98% | 100,00% |
| #M215 | ED | **T1** | 29,20 | 12187 | 5598 | 26348 | 4761 | 2562 | 6807 | 70,31% | 100,00% | 99,97% | 100,00% |
|  |  | **T2** | 49,90 | 13282 | 6055 | 28572 | 7352 | 3727 | 11591 | 67,25% | 100,00% | 100,00% | 100,00% |
| #M273 | ED | **T1** | 25,40 | 11725 | 5594 | 25179 | 6333 | 3378 | 9873 | 68,23% | 100,00% | 100,00% | 100,00% |
|  |  | **T2** | 50,00 | 13333 | 6411 | 28538 | 7474 | 3995 | 11832 | 68,21% | 100,00% | 100,00% | 100,00% |
| #M276 | ED | **T1** | 26,40 | 13292 | 6012 | 28983 | 5926 | 3171 | 9023 | 69,15% | 100,00% | 100,00% | 100,00% |
|  |  | **T2** | 50,40 | 14017 | 5919 | 33954 | 8032 | 3923 | 14289 | 69,72% | 100,00% | 100,00% | 100,00% |
| #M300 | ED | **T1** | 50,40 | 13395 | 6434 | 28565 | 8414 | 4393 | 14212 | 67,18% | 100,00% | 100,00% | 100,00% |
|  |  | **T2** | 49,80 | 13895 | 6228 | 30689 | 8085 | 4136 | 13297 | 70,21% | 100,00% | 100,00% | 100,00% |
| #M168 | HPD | **T1** | 17,90 | 12382 | 4313 | 25892 | 3384 | 1902 | 4748 | 69,32% | 100,00% | 99,59% | 100,00% |
|  |  | **T2** | 12,40 | 11067 | 3608 | 25111 | 3529 | 1755 | 5245 | 70,50% | 100,00% | 99,26% | 100,00% |
| #M182 | HPD | **T1** | 50,00 | 16314 | 4923 | 35332 | 7471 | 3038 | 11342 | 73,31% | 100,00% | 100,00% | 100,00% |
|  |  | **T2** | 50,00 | 13851 | 4604 | 32068 | 7065 | 2648 | 11942 | 79,14% | 100,00% | 99,99% | 100,00% |
| #M202 | HPD | **T1** | 50,00 | 15335 | 5151 | 34557 | 7124 | 3187 | 11193 | 70,42% | 100,00% | 100,00% | 100,00% |
|  |  | **T2** | 50,00 | 11437 | 3895 | 27367 | 6691 | 2770 | 11975 | 68,79% | 100,00% | 99,99% | 100,00% |
| #M257 | HPD | **T1** | 50,00 | 14971 | 4696 | 33982 | 7598 | 2990 | 14306 | 73,495 | 100,00% | 100,00% | 100,00% |
|  |  | **T2** | 47,60 | 13139 | 4370 | 34336 | 6980 | 2777 | 13591 | 81,61% | 100,00% | 99,99% | 100,00% |
| #M148 | PD | **T1** | 13,90 | 14295 | 5377 | 29927 | 3249 | 1961 | 4516 | 70,34% | 100,00% | 99,68% | 100,00% |
|  |  | **T2** | 14,30 | 10261 | 3646 | 23523 | 4487 | 2119 | 7114 | 68,99% | 100,00% | 99,83% | 100,00% |
| #M262 | PD | **T1** | 29,30 | 15698 | 5524 | 34564 | 5047 | 2493 | 7423 | 75,31% | 100,00% | 99,97% | 100,00% |
|  |  | **T2** | 32,50 | 12377 | 3404 | 30000 | 4843 | 1870 | 7559 | 75,47% | 100,00% | 99,54% | 100,00% |
| #M204 | PD | **T1** | 19,90 | 10586 | 3732 | 23200 | 3322 | 1769 | 4848 | 70,51% | 100,00% | 99,30% | 100,00% |
|  |  | **T2** | 18,70 | 13394 | 3947 | 31862 | 3461 | 1661 | 5342 | 74,86% | 100,00% | 98,93% | 100,00% |
| #M234 | PD | **T1** | 50,00 | 10695 | 3926 | 26370 | 6186 | 2783 | 11473 | 72,14% | 100,00% | 99,99% | 100,00% |
|  |  | **T2** | 50,00 | 12575 | 4199 | 31568 | 6549 | 2822 | 11826 | 74,03% | 100,00% | 99,99% | 100,00% |
| #M248 | PD | **T1** | 41,90 | 11321 | 3680 | 24006 | 5118 | 2235 | 7836 | 71,09% | 100,00% | 99,90% | 100,00% |
|  |  | **T2** | 49,30 | 12803 | 4045 | 27926 | 7331 | 2826 | 12321 | 69,56% | 100,00% | 99,99% | 100,00% |
| #M264 | PD | **T1** | 24,40 | 11348 | 3866 | 25202 | 3377 | 1678 | 4999 | 72,12% | 100,00% | 99,00% | 100,00% |
|  |  | **T2** | 35,00 | 12503 | 4228 | 27832 | 5786 | 2573 | 9013 | 70,68% | 100,00% | 99,98% | 100,00% |
| #M268 | PD | **T1** | 15,30 | 9829 | 3119 | 21475 | 2210 | 1133 | 3251 | 68,78% | 100,00% | 92,18% | 100,00% |
|  |  | **T2** | 21,20 | 12656 | 3965 | 27971 | 4560 | 2159 | 6704 | 71,27% | 100,00% | 99,86% | 100,00% |
| #M110 | PD | **T1** | 50,00 | 20267 | 5642 | 48740 | 8458 | 3389 | 13461 | 76,51% | 100,00% | 100,00% | 100,00% |
|  |  | **T2** | 50,00 | 17738 | 4980 | 42596 | 8561 | 3298 | 14177 | 72,99% | 100,00% | 100,00% | 100,00% |
| #M185 | CB | **T1** | 50,00 | 13978 | 4673 | 30781 | 8092 | 3253 | 13847 | 70,23% | 100,00% | 100,00% | 100,00% |
|  |  | **T2** | 48,30 | 17387 | 5921 | 38888 | 7511 | 3508 | 11370 | 70,34% | 100,00% | 100,00% | 100,00% |
| #M199 | CB | **T1** | 32,40 | 12690 | 6384 | 25786 | 4967 | 3069 | 7085 | 67,15% | 100,00% | 100,00% | 100,00% |
|  |  | **T2** | 19,45 | 12400 | 4077 | 28453 | 4842 | 2288 | 7514 | 70,96% | 100,00% | 99,92% | 100,00% |
| #M222 | CB | **T1** | 26,82 | 12046 | 3877 | 27193 | 5085 | 2281 | 7955 | 70,20% | 100,00% | 99,92% | 100,00% |
|  |  | **T2** | 22,90 | 12320 | 3812 | 28318 | 5388 | 2278 | 8693 | 70,38% | 100,00% | 99,91% | 100,00% |
| #M277 | CB | **T1** | 16,60 | 10210 | 3356 | 22579 | 3271 | 1646 | 4776 | 70,25% | 100,00% | 98,87% | 100,00% |
|  |  | **T2** | 26,10 | 11339 | 3453 | 24925 | 4048 | 1868 | 5953 | 67,80% | 100,00% | 99,53% | 100,00% |
| #M284 |  | **T1** | 31,50 | 11535 | 3563 | 25073 | 4621 | 2100 | 6863 | 71,31% | 100,00% | 99,82% | 100,00% |
|  |  | **T2** | 31,90 | 15572 | 5260 | 32973 | 5720 | 2846 | 8387 | 72,01% | 100,00% | 99,99% | 100,00% |
| #M238 | CB | **T1** | 22,70 | 16508 | 5360 | 36279 | 5527 | 2791 | 7916 | 71,66% | 100,00% | 99,99% | 100,00% |
|  |  | **T2** | 24,35 | 10252 | 3491 | 23403 | 4599 | 2151 | 7358 | 68,88% | 100,00% | 99,85% | 100,00% |
| #M249 | CB | **T1** | 28,65 | 10920 | 3713 | 24293 | 4677 | 2153 | 7397 | 68,53% | 100,00% | 99,86% | 100,00% |
|  |  | **T2** | 17,55 | 10094 | 3519 | 22422 | 3415 | 1747 | 5197 | 68,49% | 100,00% | 99,24% | 100,00% |
| #M251 | CB | **T1** | 50,00 | 14094 | 4930 | 32544 | 7937 | 3393 | 14331 | 68,98% | 100,00% | 100,00% | 100,00% |
|  |  | **T2** | 50,00 | 13188 | 4308 | 31603 | 7921 | 3083 | 14713 | 72,00% | 100,00% | 100,00% | 100,00% |

**Supplemental Table 4. Correlation between cfDNA concentration and maxVAF identified at T1 and T2.**

| **Parameter1** | **Parameter2** | **rho** | **95% CI** | **p (Spearman)** |
| --- | --- | --- | --- | --- |
| cfDNA.1 | VAF1 | 0.59 | [ 0.25, 0.80] | ***0.0060*** |
| cfDNA.1 | VAF2 | 0.49 | [ 0.10, 0.75] | ***0.0290*** |
| cfDNA.1 | diffVAF21 | -0.02 | [-0.42, 0.39] | 0.9360 |
| **cfDNA.1** | **rdiffVAF21** | **-0.16** | **[-0.53, 0.26]** | **0.5090** |
| cfDNA.2 | VAF1 | 0.51 | [ 0.13, 0.76] | ***0.0230*** |
| cfDNA.2 | VAF2 | 0.58 | [ 0.23, 0.80] | ***0.0060*** |
| cfDNA.2 | diffVAF21 | 0.39 | [-0.02, 0.69] | 0.0900 |
| **cfDNA.2** | **rdiffVAF21** | **0.18** | **[-0.24, 0.55]** | **0.4980** |
| VAF1 | diffDNA.21 | 0.16 | [-0.26, 0.53] | 0.5090 |
| VAF1 | rdiffDNA.21 | 0.02 | [-0.39, 0.42] | 0.9360 |
| VAF2 | diffDNA.21 | 0.4 | [-0.01, 0.69] | 0.0870 |
| VAF2 | rdiffDNA.21 | 0.32 | [-0.10, 0.64] | 0.1920 |
| diffVAF21 | diffDNA.21 | 0.66 | [ 0.35, 0.84] | ***0.0010*** |
| diffVAF21 | rdiffDNA.21 | 0.68 | [ 0.38, 0.85] | ***0.0000*** |
| **rdiffVAF21** | **diffDNA.21** | **0.43** | **[ 0.03, 0.71]** | **0.0560** |
| **rdiffVAF21** | **rdiffDNA.21** | **0.53** | **[ 0.16, 0.77]** | ***0.0170*** |

**Supplemental Table 5. Association of clinical characteristics of patients and cfDNA concentration.**

Association of clinical characteristics of patients and cfDNA concentration (ng per ml of plasma) at T1 (cfDNA1), at T2 (cfDNA2), and its absolute (cfDNA2-1) and relative variation T1-T2 [(cfDNA2-cfDNA1)/cfDNA1].

|  |  |  | **cfDNA1** | | **cfDNA2** | | **cfDNA2-cfDNA1** | | **cfDNA2-cfDNA1**  **cfDNA1** | |
| --- | --- | --- | --- | --- | --- | --- | --- | --- | --- | --- |
|  |  |  | **Median** | ***p value*** | **Median** | ***p value*** | **Median** | ***p value*** | **Median** | ***p value*** |
|  |  | **N** | **(Q1, Q3)** | ***KW-test*** | **(Q1, Q3)** | ***KW-test*** | **(Q1, Q3)** | ***KW-test*** | **(Q1, Q3)** | ***KW-test*** |
| **Sex** | **Male** | 24 | 15.96 (9.44, 26.59) | ***0.037*** | 29.26 (11.57, 71.41) | ***0.013*** | 4.26 (0.58, 48.83) | ***0.050*** | 0.41 (0.04, 1.99) | *0.09* |
|  | **Female** | 8 | 7.72 (5.86, 9.07) |  | 8.30 (5.89, 15.01) |  | 0.10 (-2.00, 0.69) |  | 0.01 (-0.18, 0.09) |  |
| **Smoking** | **No** | 2 | 8.02 (6.48, 9.55) | *0.168* | 7.88 (5.55, 10.20) | ***0.032*** | -0.14 (-0.93, 0.66) | ***0.041*** | -0.11 (-0.23, 0.01) | ***0.041*** |
|  | **Yes** | 10 | 7.81 (6.18, 21.18) |  | 8.41 (6.20, 22.67) |  | 0.10 (-2.18, 1.31) |  | 0.01 (-0.10, 0.10) |  |
|  | **Former** | 20 | 15.96 (11.03, 22.35) |  | 34.77 (12.54, 72.06) |  | 10.89 (1.85, 53.11) |  | 0.56 (0.07, 2.59) |  |
| **Performance status** | **0** | 15 | 16.10 (8.20, 22.90) | *0.336* | 22.75 (8.30, 48.48) | *0.895* | 0.28 (-2.28, 13.14) | *0.299* | 0.03 (-0.23, 1.46) | *0.18* |
|  | **1-2** | 17 | 11.08 (6.45, 19.13) |  | 12.58 (8.48, 67.14) |  | 2.62 (0.85, 38.08) |  | 0.41 (0.07, 1.39) |  |
| **Histology** | **Adenocarcinoma** | 25 | 12.15 (7.63, 24.00) | *0.214* | 21.43 (8.33, 67.14) | *0.167* | 2.32 (-0.07, 38.08) | *0.332* | 0.13 (-0.01, 1.87) | *0.825* |
|  | **Squamous** | 6 | 12.52 (6.11, 15.94) |  | 18.99 (5.94, 25.54) |  | 1.77 (-1.35, 7.92) |  | 0.06 (-0.15, 0.48) |  |
|  | **Other** | 1 | 138.26 (138.26, 138.26) |  | 194.35 (194.35, 194.35) |  | 56.09 (56.09, 56.09) |  | 0.41 (0.41, 0.41) |  |
| **PD-L1** | **N-Miss** | 7 |  |  |  |  |  |  |  |  |
|  | **Negative** | 15 | 14.54 (8.68, 21.59) | *0.471* | 22.44 (10.44, 55.37) | *0.956* | 3.79 (0.39, 36.22) | *0.542* | 0.54 (0.05, 2.36) | *0.108* |
|  | **Positive** | 10 | 15.88 (8.77, 66.87) |  | 22.73 (6.81, 78.59) |  | 0.94 (-2.05, 36.80) |  | 0.07 (-0.28, 0.34) |  |
| **PD-L1** | **N-Miss** | 7 |  |  |  |  |  |  |  |  |
|  | **<50%** | 17 | 14.54 (8.40, 21.80) | *0.560* | 22.44 (8.48, 66.78) | *0.907* | 3.79 (0.28, 34.36) | *0.641* | 0.36 (0.03, 1.39) | *0.244* |
|  | **≥50%** | 8 | 15.88 (10.17, 60.35) |  | 22.73 (6.21, 75.52) |  | 0.94 (-3.52, 49.70) |  | 0.10 (-0.41, 0.77) |  |
| **Extrathoracic sites** | **No** | 13 | 8.00 (6.45, 21.39) | *0.265* | 9.07 (6.60, 22.44) | *0.276* | 0.85 (-0.66, 12.48) | *0.810* | 0.11 (-0.06, 0.72) | *0.804* |
|  | **1** | 8 | 15.78 (10.72, 23.78) |  | 18.99 (7.83, 75.52) |  | 4.86 (-0.77, 49.70) |  | 0.24 (-0.05, 0.90) |  |
|  | **>1** | 11 | 15.81 (10.34, 40.90) |  | 32.92 (17.64, 65.59) |  | 4.04 (1.45, 25.68) |  | 0.17 (0.06, 3.26) |  |
| **N metastatic sites** | **0-1** | 18 | 11.82 (7.49, 23.35) | *0.761* | 21.08 (7.02, 67.56) | *0.820* | 2.47 (-0.11, 45.20) | *0.569* | 0.41 (-0.01, 1.23) | *0.649* |
|  | **2-4** | 14 | 14.99 (8.70, 21.13) |  | 24.17 (12.43, 59.02) |  | 2.62 (-1.31, 13.86) |  | 0.10 (-0.14, 2.30) |  |
| **Treatment lines** | **1** | 9 | 20.25 (11.08, 52.57) | *0.046* | 43.96 (12.53, 90.65) | *0.098* | 34.36 (-2.83, 47.57) | *0.722* | 0.41 (-0.35, 1.87) | *0.917* |
|  | **>1** | 23 | 11.50 (6.17, 18.75) |  | 20.73 (7.44, 44.82) |  | 2.32 (-0.16, 10.89) |  | 0.11 (-0.02, 0.99) |  |

**Supplemental Table 6. Association of clinical characteristics of patients and NGS results.**

Association of clinical characteristics of patients and of the value at T1 and T2 of the highest VAF (maxVAF) among all genetic alterations at baseline, and its absolute (VAF2-1) and relative variation T1-T2 [(VAF2-VAF1)/VAF1].

|  |  |  | **maxVAF1** | | **maxVAF2** | | **maxVAF2-maxVAF1** | | **maxVAF2-maxVAF1**  **maxVAF1** | |
| --- | --- | --- | --- | --- | --- | --- | --- | --- | --- | --- |
|  |  |  |  | ***p value*** |  | ***p value*** |  | ***p value*** |  | ***p value*** |
|  |  | **N** | **Median (Q1, Q3)** | ***KW-test*** | **Median (Q1, Q3)** | ***KW-test*** | **Median (Q1, Q3)** | ***KW-test*** | **Median (Q1, Q3)** | ***KW-test*** |
| **Sex** | **Male** | 18 | 6.72 (1.94, 28.52) | *0.226* | 6.87 (1.45, 32.15) | *0.183* | 0.34 (-2.13, 3.95) | *0.586* | 0.13 (-0.32, 0.49) | *0.856* |
|  | **Female** | 7 | 3.39 (1.33, 4.93) |  | 1.74 (1.16, 5.60) |  | 0.03 (-0.63, 0.71) |  | 0.05 (-0.09, 0.42) |  |
| **Smoking** | **No** | 2 | 6.78 (3.75, 9.80) | *0.858* | 6.75 (4.24, 9.25) | *0.476* | -0.03 (-0.55, 0.48) | *0.243* | 0.64 (0.28, 1.01) | *0.379* |
|  | **Yes** | 9 | 3.39 (1.93, 5.53) |  | 2.61 (1.41, 7.39) |  | -0.19 (-5.04, 0.43) |  | -0.10 (-0.44, 0.13) |  |
|  | **Former** | 14 | 6.72 (2.12, 28.47) |  | 13.29 (1.02, 33.73) |  | 0.94 (-1.21, 5.26) |  | 0.18 (-0.08, 0.49) |  |
| **Performance status** | **0** | 12 | 4.93 (1.93, 12.45) | *0.913* | 4.33 (1.51, 12.12) | *0.744* | 0.47 (-1.80, 2.43) | *1.000* | 0.08 (-0.45, 0.88) | *0.828* |
|  | **1-2** | 13 | 3.92 (1.17, 28.50) |  | 4.06 (1.41, 29.84) |  | 0.07 (-1.06, 1.44) |  | 0.10 (-0.11, 0.46) |  |
| **Histology** | **Adenocarcinoma** | 19 | 5.53 (1.92, 25.71) | *0.357* | 4.83 (1.16, 20.90) | *0.375* | 0.03 (-2.33, 2.25) | *0.256* | 0.05 (-0.38, 0.33) | *0.375* |
|  | **Squamous** | 5 | 2.00 (0.73, 2.70) |  | 1.73 (1.41, 4.06) |  | 1.00 (0.52, 1.36) |  | 0.50 (0.04, 1.37) |  |
|  | **Other** | 1 | 28.53 (28.53, 28.53) |  | 37.93 (37.93, 37.93) |  | 9.40 (9.40, 9.40) |  | 0.33 (0.33, 0.33) |  |
| **PD-L1** | **N-Miss** | 6 |  |  |  |  |  |  |  |  |
|  | **Negative** | 11 | 3.92 (1.94, 28.45) | *0.563* | 4.06 (2.08, 33.46) | *0.509* | 1.36 (0.25, 3.38) | *0.032* | 0.16 (0.07, 0.87) | *0.021* |
|  | **Positive** | 8 | 9.97 (4.63, 30.30) |  | 6.87 (1.45, 13.83) |  | -1.68 (-7.67, -0.13) |  | -0.21 (-0.54, -0.05) |  |
| **PD-L1** | **N-Miss** | 6 |  |  |  |  |  |  |  |  |
|  | **<50%** | 13 | 3.92 (1.93, 28.50) | *0.661* | 4.06 (1.74, 32.92) | *0.539* | 0.52 (-0.19, 2.22) | *0.161* | 0.13 (-0.10, 0.50) | *0.096* |
|  | **≥50%** | 6 | 9.97 (5.93, 24.60) |  | 6.87 (1.65, 11.05) |  | -1.68 (-4.36, -0.24) |  | -0.20 (-0.70, 0.02) |  |
| **Extrathoracic sites** | **No** | 10 | 2.70 (1.03, 5.13) | *0.272* | 1.64 (0.92, 3.30) | *0.242* | 0.11 (-0.49, 0.86) | *0.871* | 0.11 (-0.25, 0.38) | *0.834* |
|  | **1** | 4 | 17.83 (5.49, 35.10) |  | 21.38 (3.77, 42.70) |  | 1.13 (-0.55, 4.02) |  | 0.05 (-0.05, 0.12) |  |
|  | **>1** | 11 | 6.32 (2.31, 31.11) |  | 8.91 (3.33, 20.90) |  | 0.52 (-2.75, 2.25) |  | 0.19 (-0.28, 0.97) |  |
| **N. metastatic sites** | **0-1** | 13 | 3.92 (1.93, 23.03) | *0.703* | 1.74 (0.75, 32.92) | *0.550* | 0.07 (-0.59, 2.22) | *0.913* | 0.05 (-0.30, 0.16) | *0.301* |
|  | **2-4** | 12 | 5.32 (1.73, 29.80) |  | 8.15 (2.39, 20.48) |  | 0.76 (-2.19, 1.85) |  | 0.35 (-0.20, 1.27) |  |
| **Treatment lines** | **1** | 7 | 23.03 (9.18, 31.12) | *0.034* | 11.76 (6.48, 33.88) | *0.146* | -1.06 (-4.46, 5.38) | *0.545* | -0.08 (-0.47, 0.42) | *0.545* |
|  | **>1** | 18 | 2.70 (0.84, 6.93) |  | 2.17 (0.98, 16.89) |  | 0.29 (-0.49, 2.03) |  | 0.11 (-0.25, 0.65) |  |

**Supplemental Table 7. Optimal cutpoint performance.**

|  | **full sample** | | **bootstraping** | | |
| --- | --- | --- | --- | --- | --- |
| **Predictor** | **optimal cutpoint** | **accuracy (95%CI** | **median in-bag cut-point (95%CI)** | **median in-bag accuracy (95%CI)** | **median out-of-bag accuracy (95%CI)** |
| **cfDNA1** | 12.15 | 0.69 (0.50, 0.84) | 12.15 (8.95, 34.38) | 0.75 (0.62, 0.87) | 0.64 (0.36, 0.82) |
| **cfDNA2** | 22.75 | 0.81 (0.64, 0.93) | 22.75 (12.4, 67.12) | 0.88 (0.78, 0.94) | 0.77 (0.57, 0.92) |
| **cfDNA2-cfDNA1** | 3.79 | 0.88 (0.71, 0.96) | 3.79 (3.79, 12.48) | 0.94 (0.84, 1) | 0.89 (0.70, 1) |
| **cfDNA2-cfDNA1**  **cfDNA1** | 0.17 | 0.78 (0.60, 0.91) | 0.17 (0.07, 0.54) | 0.78 (0.62, 0.94) | 0.79 (0.60, 0.93) |
| **maxVAF1** | 4.32 | 0.80 (0.56, 0.93) | 4.32 (0.73, 33.71) | 0.56 (0.32, 0.84) | 0.80 (0.25, 0.78) |
| **maxVAF2** | 0.84 | 0.80 (0.59, 0.93) | 1.73 (0.84, 21.75) | 0.44 (0.18, 0.71) | 1 (0.50, 1) |
| **maxVAF2-maxVAF1** | 0.52 | 0.80 (0.59, 0.93) | 0.52 (0.52, 3.07) | 0.72 (0.56, 0.88) | 0.64 (0.43, 0.86) |
| **maxVAF2-maxVAF1**  **maxVAF1** | 0.71 | 0.84 (0.64, 0.95) | 0.71 (0.71, 1.62) | 0.92 (0.76, 1) | 0.86 (0.62, 1) |
