# Supplementary figures and images for: Longitudinal liquid biopsy anticipates hyperprogression and early death in advanced non-small cell lung cancer patients treated with immune checkpoint inhibitors
Source: Br J Cancer. 2022 Sep 29;127(11):2034–42. doi: 10.1038/s41416-022-01978-1 (PMC9681746; doi:10.1038/s41416-022-01978-1)

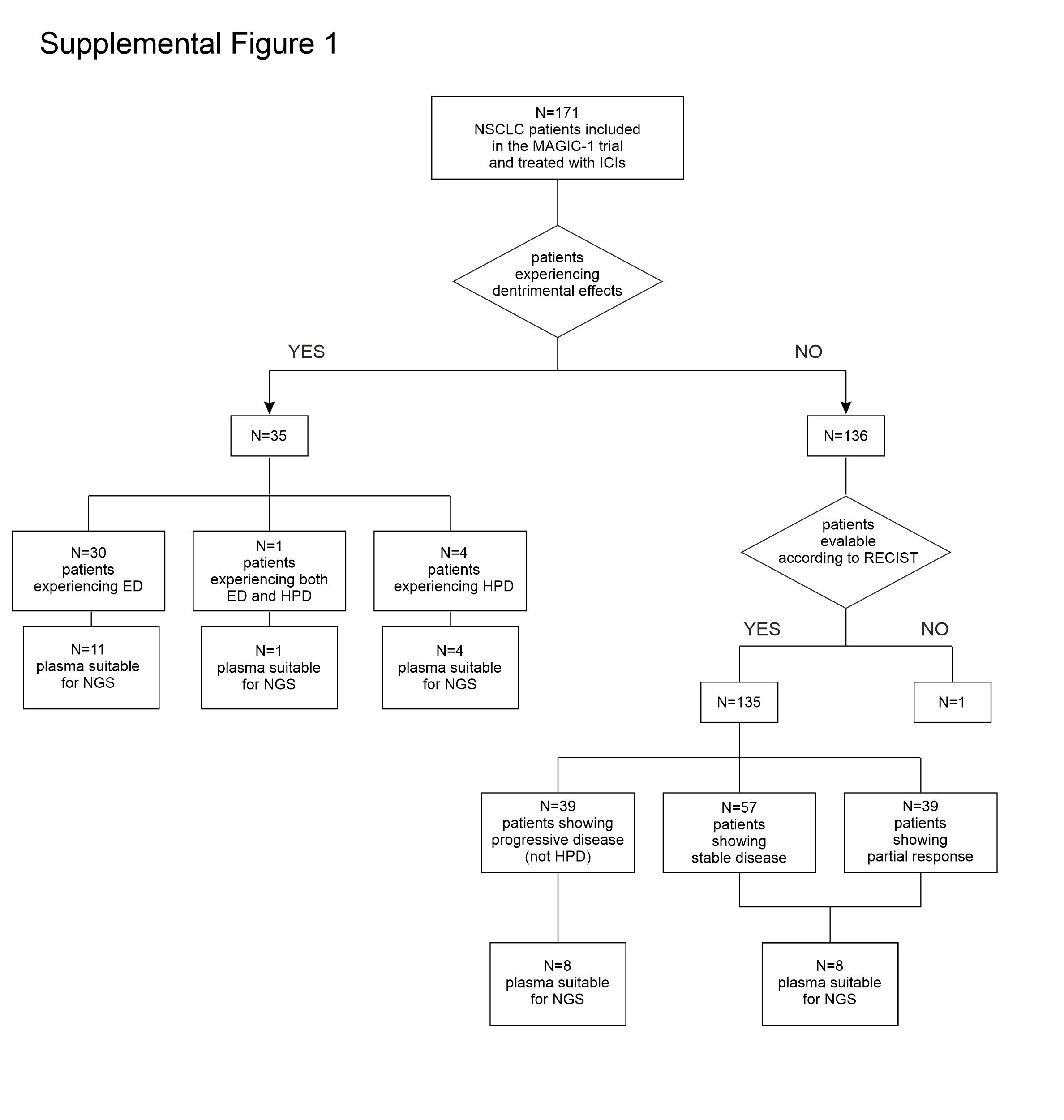

Supplement: Supplementary file 2 — Supplemental Figure 1 [file 41416_2022_1978_MOESM2_ESM.jpg]

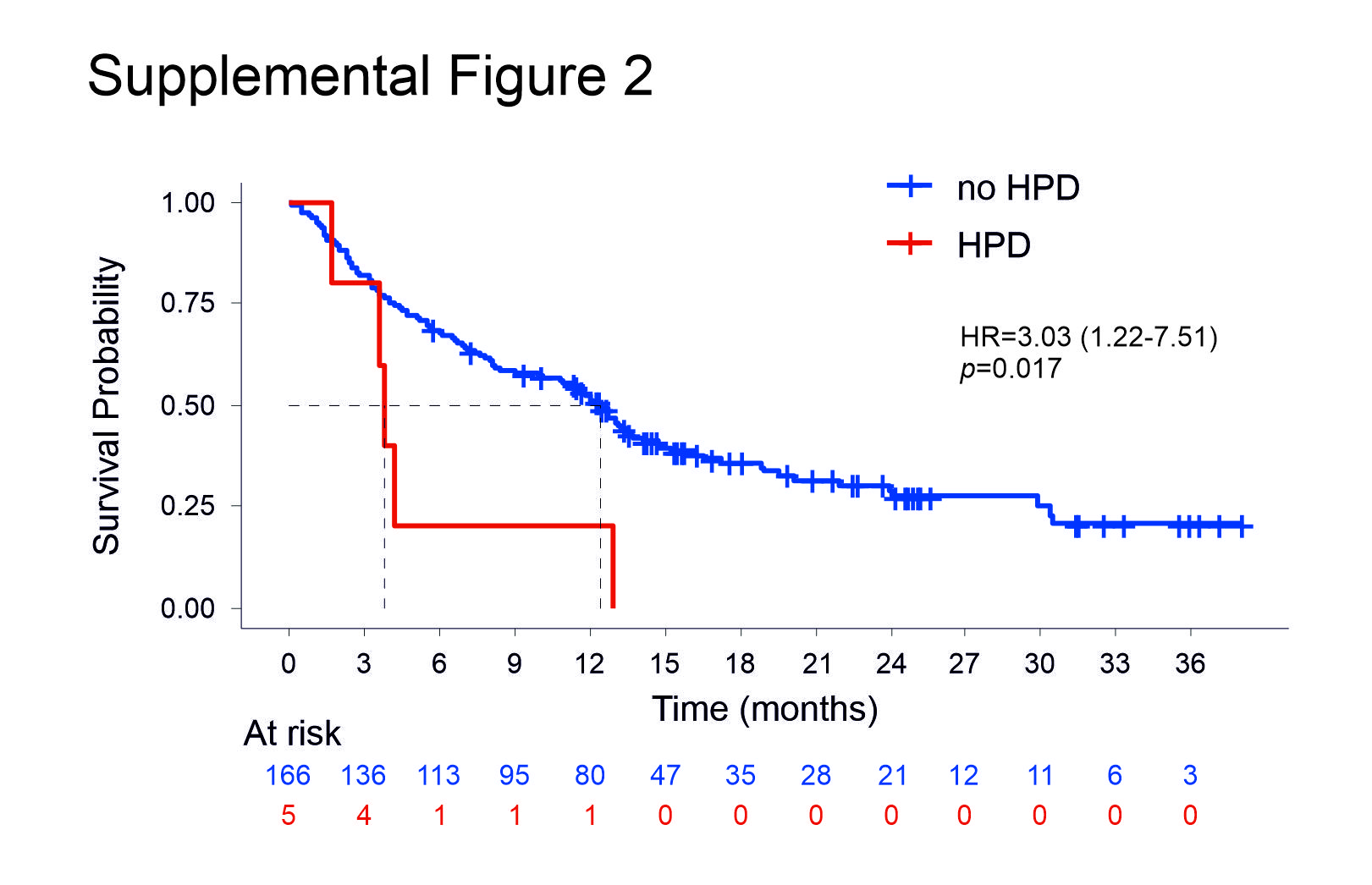

Supplement: Supplementary file 3 — Supplemental Figure 2 [file 41416_2022_1978_MOESM3_ESM.jpg]

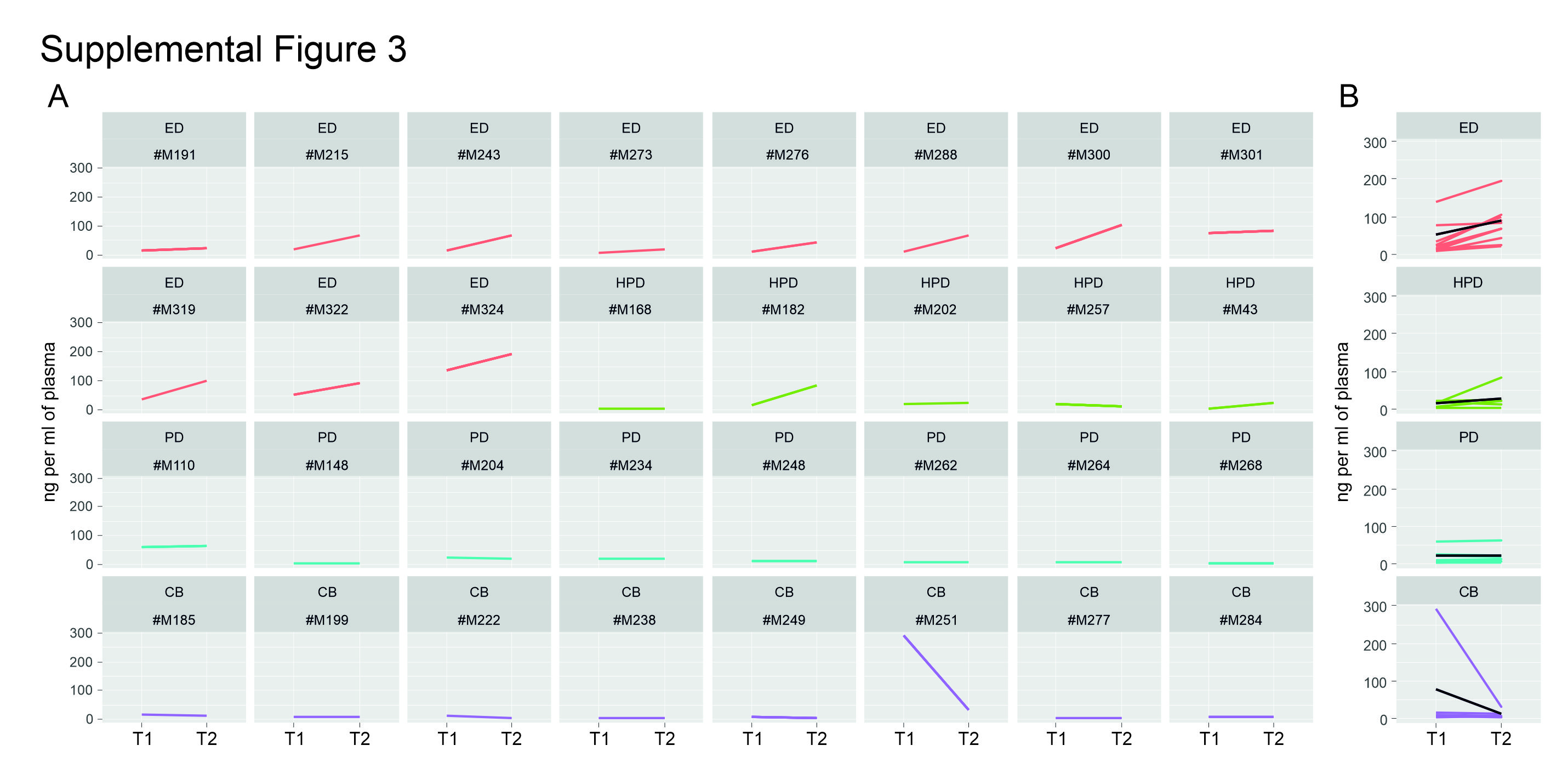

Supplement: Supplementary file 4 — Supplemental Figure 3 [file 41416_2022_1978_MOESM4_ESM.jpg]

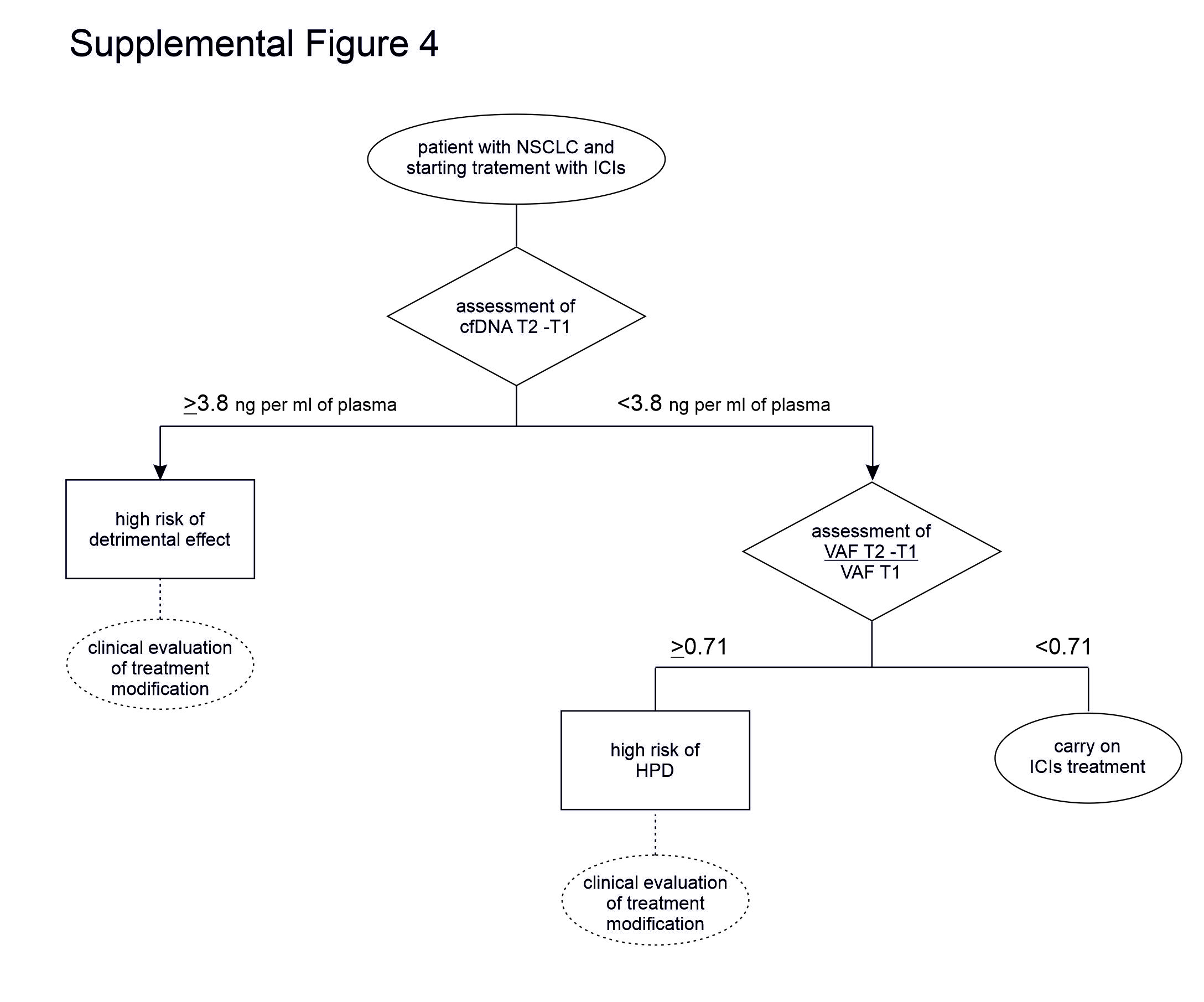

Supplement: Supplementary file 5 — Supplemental Figure 4 [file 41416_2022_1978_MOESM5_ESM.jpg]
